# Involvement of Inflammation and Adverse Vascular Remodelling in the Blood Pressure Raising Effect of Repeatedly Heated Palm Oil in Rats

**DOI:** 10.1155/2012/404025

**Published:** 2012-06-21

**Authors:** Chun-Yi Ng, Yusof Kamisah, Othman Faizah, Zakiah Jubri, Hj Mohd Saad Qodriyah, Kamsiah Jaarin

**Affiliations:** ^1^Department of Pharmacology, Faculty of Medicine, Universiti Kebangsaan Malaysia, 50300 Kuala Lumpur, Malaysia; ^2^Department of Anatomy, Faculty of Medicine, Universiti Kebangsaan Malaysia, 50300 Kuala Lumpur, Malaysia; ^3^Department of Biochemistry, Faculty of Medicine, Universiti Kebangsaan Malaysia, 50300 Kuala Lumpur, Malaysia

## Abstract

Oil thermoxidation during deep frying generates harmful oxidative free radicals that induce inflammation and increase the risk of hypertension. This study aimed to investigate the effect of repeatedly heated palm oil on blood pressure, aortic morphometry, and vascular cell adhesion molecule-1 (VCAM-1) expression in rats. Male Sprague-Dawley rats were divided into five groups: control, fresh palm oil (FPO), one-time-heated palm oil (1HPO), five-time-heated palm oil (5HPO), or ten-time-heated palm oil (10HPO). Feeding duration was six months. Blood pressure was measured at baseline and monthly using tail-cuff method. After six months, the rats were sacrificed and the aortic arches were dissected for morphometric and immunohistochemical analyses. FPO group showed significantly lower blood pressure than all other groups. Blood pressure was increased significantly in 5HPO and 10HPO groups. The aortae of 5HPO and 10HPO groups showed significantly increased thickness and area of intima-media, circumferential wall tension, and VCAM-1 than other groups. Elastic lamellae were disorganised and fragmented in 5HPO- and 10HPO-treated rats. VCAM-1 expression showed a significant positive correlation with blood pressure. In conclusion, prolonged consumption of repeatedly heated palm oil causes blood pressure elevation, adverse remodelling, and increased VCAM-1, which suggests a possible involvement of inflammation.

## 1. Introduction

The practice of reusing vegetable oils several times for deep frying before disposing them is quite common among Malaysians. It is thought to be a way to cut the expense. Such practice might be detrimental. However, general public awareness about this is only at moderate level [1]. Deep fried foods have been becoming more popular in daily diet, especially in this modern fast-paced lifestyle. Heating the vegetable oils to a high level of temperature, that is, approximately 160–180°C, also exposes them to the air and moisture at the same time, in which the oils will undergo a complex series of physical and chemical deterioration known as oil thermoxidation. This oxidative deterioration affects the chemical compositions of the vegetable oils by saturating its fatty acids and generating reactive oxygen species (ROS) which are potential in causing deleterious effects on the normal function of endothelial cells [2] and increasing risk of hypertension [3, 4].

Due to their unpaired shell electron, ROS are highly damaging to cells and therefore recognised to be a major cause of endothelial dysfunction and vascular inflammation [5–7]. Pathogenesis of hypertension might be attributed to inflammation [8]. Several reports documented that inflammation may play a pivotal role in the initiation as well as progression of hypertension [9, 10]. Endothelial cells which line the intimal surface of blood vessel and maintain the integrity of the vascular system are the primary target of immunological attack in inflammatory diseases. Endothelial dysfunction is manifested by altered anti-inflammatory properties of the endothelium, impaired modulation of vascular growth, leukocyte adhesion, dysregulation of vasomotion, and smooth muscle cell proliferation [11–13], which may play a major role in the development of high blood pressure. Vascular cell adhesion molecule-1 (VCAM-1) is one of the endothelial cell adhesion molecules that mediate leukocytes binding. The increased expression of VCAM-1 on endothelial cells is a common process in response to inflammation [14], and it is recognised as an important cardiovascular risk marker [15, 16]. Nevertheless, ROS also stimulate expression of adhesion and chemotactic molecules, which promote uptake of inflammatory cells into the vessel wall [5]. Previous works found elevated level of soluble VCAM-1 in hypertensive subjects [17–19].

Palm oil, which contains both saturated fatty acids (SFA) and monounsaturated fatty acids (MUFA) at almost similar levels [20], is popular in the food industry as well as in family kitchen due to its oxidative stability. It is a commonly used vegetable oil in Malaysia. It has been previously demonstrated that consumption of repeatedly heated palm oil causes a significant elevation in blood pressure [4]. We believe that the ROS and other harmful oxidation products present in the repeatedly heated vegetable oils may induce inflammation in vascular system. The present study aimed to investigate the possible role of inflammation in blood pressure elevation after the prolonged intake of repeatedly heated palm oil in blood vessel as well as the vascular morphometric alterations.

## 2. Materials and Methods

### 2.1. Experimental Design

Thirty adult male Sprague-Dawley rats (*n* = 30) aged three months, weighing 200–280 g were obtained from the Laboratory Animal Resource Unit, Universiti Kebangsaan Malaysia. The handling and experimental protocols were approved by the Universiti Kebangsaan Malaysia Animal Ethics Committee. The animals were housed in stainless-steel cages and kept at room temperature of 27°C ± 2°C with a 12-hour light cycle at the Pharmacology Department Animal House. All rats had free access to food and tap water throughout the experiment. The animals were acclimatised for one week, prior to administration of test diets. The rats were divided into five groups comprising six animals each and given the following course of diet: (i) basal diet without any addition of oil (as control) or basal diet fortified with 15% weight/weight (w/w), (ii) fresh palm oil (FPO) as described earlier by Owu et al. [21], (iii) one-time-heated palm oil (1HPO), (iv) five-time-heated palm oil (5HPO), or (v) ten-time-heated palm oil (10HPO) for six months. Body weight and blood pressure were determined before the treatment and at monthly intervals. At the end of the study, rats were sacrificed and aortic arches were excised and processed according to the routine histological procedures for histological and immunohistochemical examination.

### 2.2. Preparation of Palm Oil Diets

Commercially purchased palm oil (Cap Buruh, Lam Soon Edible Oil, Kuala Lumpur, Malaysia) was used in fresh state or heated once, five times, and ten times, according to the modified method of Owu et al. [21]. The heating process involved using 2.5 L of the oil to fry 1 kg of sweet potatoes in a stainless-steel wok at about 180°C for 10 min. The heated oil was cooled for five hours, and then the entire frying process was repeated with a fresh batch of sweet potatoes. The process was repeated four, and nine times to obtain the five- and ten-times-heated-oil respectively. No replenishment of fresh oil was done between batches to make up for the loss due to uptake of the oil by the frying material. Standard rat chow (Gold Coin, Kepong, Malaysia) was ground and formulated by mixing 15% (w/w) of respective oils prepared. The pellets were reformed and dried in an oven at 80°C overnight.

### 2.3. Measurement of Blood Pressure

Systolic blood pressure of rats was measured by the tail-cuff method using PowerLab data acquisition systems (ADI Instruments, NSW, Australia) after warming the rats for 10 minutes. Five readings were obtained from each rat and then averaged.

### 2.4. Aortic Morphometry

Aortic arches were embedded in Paraplast Plus (Sigma-Aldrich, St. Louis, MO, USA), and 5 *μ*m cuts were accomplished (LEICA RM2235, Walldorf, Germany). Aortic sections were stained with Verhoeff-Van Gieson to identify elastic fibres and smooth muscle cells. Digital images of aortic sections were acquired (JPEG format, 24-bit colour, 2560 × 1920 pixels) with a MicroPublisher 5.0 RTV camera (Q Imaging, Surrey, BC, Canada) and a Nikon Eclipse 80i microscope (Nikon Corporation, Tokyo, Japan) and analysed with the software Image-Pro Plus version 7.0 (Media Cybernetics, Silver Spring, MD, USA). Morphometric measurements, which included intima-media thickness (IMT), intima-media area (IMA), lumen diameter, lamellar units, circumferential wall tension (CWT), and tensile stress (TS), were done according to the method described by Fernandes-Santos et al. [22].

Briefly, four measurements of IMT per image were obtained at 0°, 90°, 180°, and 270° by drawing a line across the tunica intima and media. The measurements were averaged to get the value corresponding to the single image. Lumen area (*a*) was estimated by drawing a line over the circle delimited by the inner face of the intima layer. Then by using the values of *a*, the lumen diameter (*d*) was calculated as $d=(2\sqrt{a})/\pi$, where *a* is expressed in mm^2^ and *π* is 3.14. The mean cross-sectional area of the tunica intima and tunica media (intima-media area, IMA) was calculated as IMA = [*π*(*d*/2+  IMT)^2^]−[*π*(*d*/2)^2^]. The number of elastic fibres lamellae (lamellar units) in the tunica media was counted. CWT was calculated as CWT = MSBP × (*d*/2), where CWT was expressed in dyne/cm, MSBP (mean systolic blood pressure) as dynes/cm^2^, and *d* (lumen diameter) in cm. TS was calculated as TS = CWT/IMT. It was expressed in dyne/cm^2^ and IMT in cm.

### 2.5. Immunohistochemical Study of VCAM-1

Aortic sections (5 *μ*m) cuts were accomplished and adhered to polylysine glass slides (Polysine, Thermo Scientific, Braunschweig, Germany). After deparaffinised and hydrated gradually, the sections were rinsed and subjected to microwave antigen retrieval in sodium citrate buffer (10 mM sodium citrate, 0.05% Tween 20, pH 6.0). After blocking endogenous peroxidase and nonspecific background staining, the aortic sections were then incubated with anti-VCAM-1 antibody (1 : 100, sc-8304, Santa Cruz Biotechnology, CA, USA) at room temperature for an hour. After washing, the reaction was amplified with a micropolymeric labelling technology (UltraVision Quanto Detection System HRP DAB, Thermo Fisher Scientific, Fremont, CA, USA). Antibody binding was visualised with diaminobenzidine. Sections were then counterstained with haematoxylin.

VCAM-1 immunostaining was quantified as described by Moraes-Teixeira et al. [23]. Briefly, tunica intima boundary was delimited by drawing a line over it using an irregular “area of interest” tool. Inside the delimited tunica intima, VCAM-1 immunostaining was selected and segmented into a new binary image, where white colour represented immunostaining and black colour represented unstained area. The percentage of area that was occupied by white colour was quantified using the image histogram tool [24]. VCAM-1 immunostaining was expressed as the percentage of tunica intima area (%). Measurements were obtained from five nonconsecutive aortic sections from each animal.

### 2.6. Statistical Analysis

All results were expressed as mean ± SEM. Normality of data was determined using Kolmogorov-Smirnov test. Paired Student's *t-*test was used to compare pre- and posttreatment data. The data among groups were analysed using one-way analysis of variances (ANOVA) followed by Tukey's Honestly Significant Differences (HSD) post-hoc test. Correlation between blood pressure and VCAM-1 density was analysed using Pearson's correlation test for all the animals irrespective of treatment groups. A value of *P* < 0.05 was considered as statistically significant. All statistical analyses were performed using the SPSS version 14.0 software (SPSS Inc., Chicago, IL, USA).

## 3. Results

### 3.1. Body Weight and Food Intake

There was a significant increase (*P* < 0.05) in body weight at the end of this study in all groups. However, the body weight gain and final body weight did not significantly differ among the groups. There was no significant difference in the weekly food intake in all study groups as well (Table 1).

### 3.2. Blood Pressure

**Figure 1:**
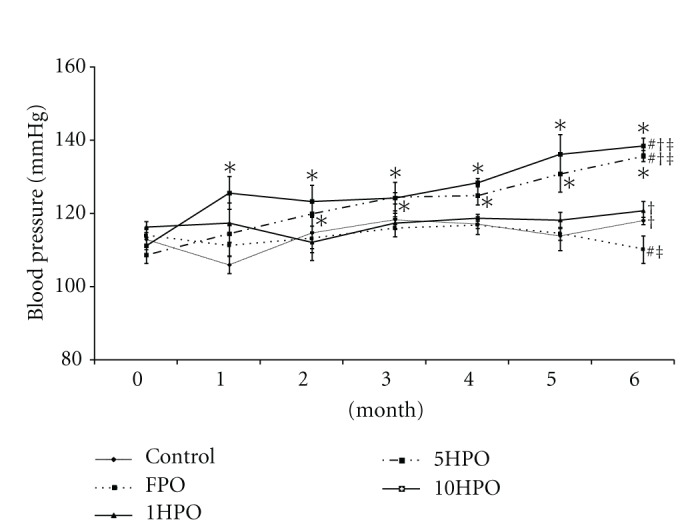
Blood pressure in all groups during the study period. Data are expressed as mean ± SEM. FPO, fresh palm oil: 1HPO, one-times-heated palm oil: 5HPO five-time-heated palm oil; 10HPO ten-time-heated palm oil. *P < 0.05 between pre- and posttreatment values for the same group; ^#^ *P* < 0.05 versus control; ^†^ *P* < 0.05 versus FPO; ^‡^ *P* < 0.05 versus 1HPO.

By the end of the study, there was a significant increase (*P* < 0.05) in blood pressure in rats fed 5HPO or 10HPO along and at the end of the study, which was observed as early as after the first month of diet administration. Rats fed 5HPO or 10HPO showed a significant increase (*P* < 0.05) in blood pressure compared to the control, FPO, and 1HPO groups. On the other hand, the blood pressure of the rats fed basal diet (control), FPO, or 1HPO did not change significantly throughout the experiment. However, we found that the rats fed FPO showed significantly lower blood pressure at the final month compared to all experimental groups (Figure 1).

### 3.3. Aortic Morphometry

Aortic sections from rats fed 5HPO or 10HPO showed significant increase (*P* < 0.05) in IMT compared to control, FPO, and 1HPO groups. Aortic IMA from 5HPO and 10HPO groups were also significantly greater (*P* < 0.05) than the control, FPO, and 1HPO groups. However, lumen diameter and elastic lamellar units did not differ significantly among the groups. With increased IMT and IMA but unaltered lumen diameter, a hypertrophic outward remodelling was indicated in the 5HPO and 10HPO groups. CWT was increased significantly (*P* < 0.05) in rats fed 5HPO or 10HPO compared to the control, FPO, and 1HPO groups. We did not observe significant difference in CWT between the control, FPO, and 1HPO groups. There were no significant differences in TS among the groups (Table 2).

**Figure 2:**
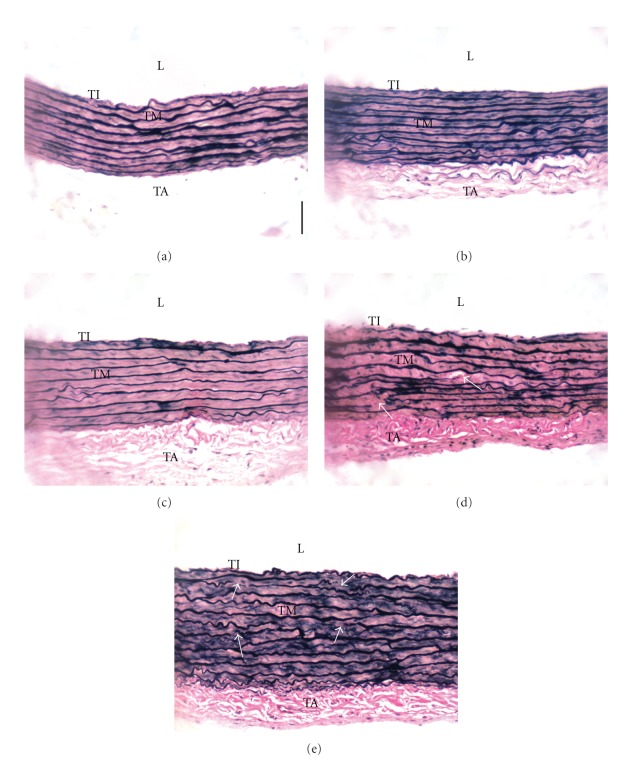
Photomicrographs of aortic sections stained with Verhoeff-Van Gieson. Groups are as follows: (a) control rats; rats fed, (b) fresh (FPO), (c) one-time-heated (1HPO), (d) five-time-heated (5HPO), or (e) ten-time-heated palm oil (10HPO). Thickened tunica media is observed in 5HPO and 10HPO groups [(d) and (e)], with an increased interlamellar space when compared to the control and FPO groups [(a) and (b)]. Disorganisation and fragmentation of the elastic lamellae were also observed in 5HPO and 10HPO (arrow, (d) and (e)). L: lumen; TI: tunica intima; TM: tunica media; TA: tunica adventitia. Same magnification is applied to all pictures (×200). Calibration bar = 50 *μ*m.

Aortic architecture in rats fed 5HPO or 10HPO were observed and characterised by an increase in interlamellar space in the tunica media when compared to the control and FPO groups. In addition, the elastic lamellae in 5HPO and 10HPO groups were observed to be disorganised and fragmented (arrow, Figures 2(d) and 2(e)). On the other hand, the aortic structure in FPO, and 1HPO groups did not show much remarkable difference than the control (Figures 2(a)–2(c)).

### 3.4. Expression of VCAM-1

**Figure 3:**
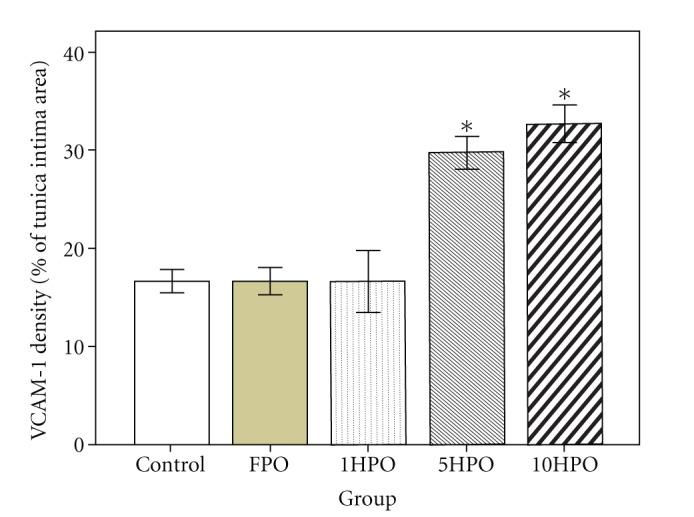
Endothelial VCAM-1 expression in rats. Data are expressed as mean ± SEM. FPO, fresh palm oil; 1HPO, one-time-heated palm oil; 5HPO, five-time-heated palm oil; 10HPO, ten-time-heated palm oil. *P < 0.05 versus control, FPO, and 1HPO.

**Figure 4:**
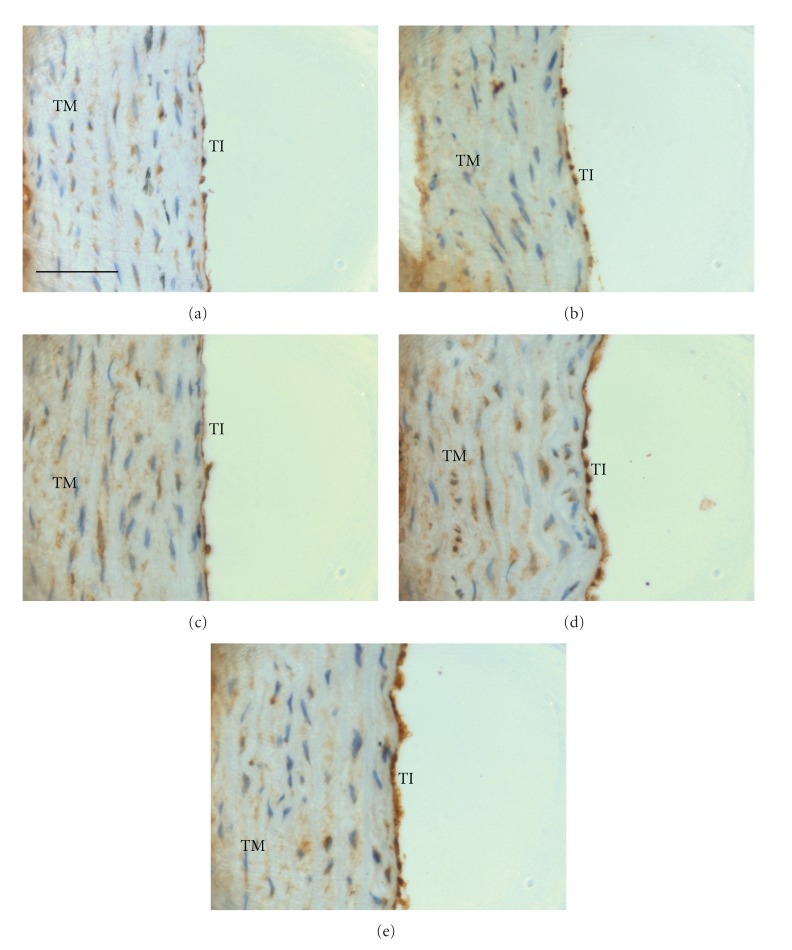
Photomicrographs of aortic sections showing immunostaining for VCAM-1. Groups are as follows: (a) control rats; rats fed (b) fresh (FPO), (c) one-time-heated (1HPO), (d) five-time-heated (5HPO), or (e) ten-time-heated palm oil (10HPO). Aortic sections from rats fed 5HPO and 10HPO showed an intense staining of VCAM-1 on the tunica intima [(d) and (e)]. In contrast, aortic sections from rats fed FPO and 1HPO showed relatively little staining of VCAM-1 [(b) and (c)] compared to control (a). TI: tunica intima; TM: tunica media. Same magnification is applied to all pictures (×400). Calibration bar = 50 *μ*m.

Positive immunostaining for VCAM-1 was observed in the endothelial cells. The aortic VCAM-1 expression was found to be significantly higher (*P* < 0.05) in rats fed 5HPO or 10HPO than the control, FPO, and 1HPO groups (Figure 3). As shown in Figures 4(a)–4(c), little VCAM-1 immunostaining was observed in the aortic sections of rats fed FPO or 1HPO when compared to the control. On the other hand, the aortic VCAM-1 expression on tunica intima was distinctly denser in the rats that fed 5HPO or 10HPO when compared to the control (Figures 4(d) and 4(e)).

### 3.5. Correlation between Blood Pressure and VCAM-1 Expression

**Figure 5:**
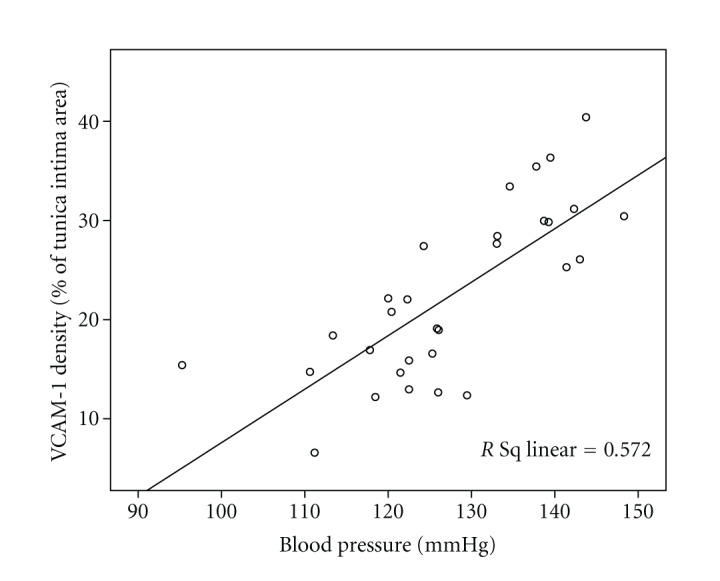
Correlation between blood pressure and endothelial VCAM-1 expression in rats.

There was a significant positive relationship (*r* = 0.757, *P* < 0.001) between systolic blood pressure and aortic VCAM-1 expression (Figure 5).

## 4. Discussion

This study was carried out to ascertain the involvement of inflammation in blood pressure elevation after consumption of heated palm oil. We postulated that heating the palm oil repeatedly would generate harmful ROS and hence induce inflammation and endothelial dysfunction.

In the present study, we observed a significant increase in blood pressure in the rats fed 5HPO or 10HPO compared to the control and rats fed FPO or 1HPO. This significant increase in blood pressure was in agreement with a previous study [4] showing prolonged intake of repeatedly heated palm oil increased blood pressure. Osim et al. [25] also reported that the oxidised oil-fed group had a greater rise in blood pressure than the fresh oil-fed group. A study carried out by Soriguer et al. [3] showed that the risk of hypertension was positively correlated with the consumption of polar compounds that were present in the cooking oil. Repeatedly heating makes the oil more susceptible to lipid peroxidation [26], which also reduces the vitamin E constituents such as *α*-tocopherol, *α*-tocotrienol, and *γ*-tocotrienol in palm and soy oils [27]. Consumption of repeatedly heated palm oil in postmenopausal state may contribute to the development of atherosclerosis because of increased lipid peroxidation [28]. Therefore, the deleterious effect of prolonged intake of 5HPO and 10HPO on blood pressure that we observed might be contributed by the overproduction of ROS that causes vascular inflammation and impairs the endothelial function. Oxidative stress, due to over-production of ROS, exerts endothelial dysfunction which plays a key role in pathogenesis of hypertension. A study done by Chan et al. [29] demonstrated that the increased levels of ROS may contribute to oxidative stress and hypertension in rats.

Blood pressure in the rats fed FPO or 1HPO did not show any remarkable change throughout the experiment. In fact, interestingly, we found that rats fed FPO even showed a significantly lower blood pressure after six months compared to the control. This shows that palm oil did not only prevent the increase of blood pressure but also had a tendency to lower it. Palm oil is rich in natural antioxidants like tocotrienol which have beneficial effect on oxidative stress associated with hypertension [30]. Medeiros et al. [31] also demonstrated that the long-term intake of palm oil had beneficial effect in reducing blood pressure in spontaneously hypertensive rats. Free radical scavenging antioxidants in palm oil serve to protect endothelial cells against oxidative injury and thus improve endothelial functions [32]. Furthermore, the blood pressure lowering mechanism of palm oil may also involve the beneficial alteration in endothelium-derived factors [33]. The present finding provides further support to the cardiovascular protective effect of palm oil.

From morphometric aspects, we observed that prolonged consumption of 5HPO or 10HPO resulted in significantly increased thickness (IMT) and area (IMA) of intima-media of the aortic wall in rats compared to those fed basal diet, FPO, or 1HPO. This finding suggests that prolonged consumption of heated oils causes vascular hypertrophic remodelling. IMT thickening and vascular architectural changes are commonly associated with high blood pressure [34]. Therefore, IMT is a marker of coronary disease [35]. Albeit increased IMT, the number of elastic lamellae did not differ among the groups, which might suggest that the IMT thickening is due to vascular smooth muscle cells hypertrophy, as indicated by increased interlamellar space. We believe that the ROS present in the heated oils may play a role in this remodelling. Previously, we observed a reduction in nitric oxide (NO) level following administration of repeatedly heated oil in rats [36]. Increased ROS generation may contribute to endothelial dysfunction by decreasing the bioavailability of NO which functions to reduce cellular proliferation. Hypertension is accompanied by an altered biochemical environment between the factors that act as cell growth promoters and the factors that reduce cellular proliferation [37]. This may lead to an imbalance between the rate of growth and death, causing wall hypertrophy. Nakaki and Kato [38] also reported that vascular remodelling could be due to decreased NO and high blood pressure. Further, TS (which is the tension per unit of thickness and acts perpendicularly to the wall) did not differ among the groups, maybe due to the IMT thickening. On the other hand, there was no significant difference of lumen diameter among the groups. This may suggest that the increased CWT that we observed in rats fed 5HPO or 10HPO could be due to the elevated blood pressure in them. CWT is the force that acts in longitudinal and circumferential directions to oppose the distending effects of blood pressure. High blood pressure increases CWT, in which further predisposing the vascular wall to damage and impairing its normal functions [39].

Prolonged consumption of 5HPO or 10HPO significantly increased the aortic endothelial expression of VCAM-1 in rats when compared to the control, FPO, and 1HPO groups. FPO that did not induce VCAM-1 expression might be due to its rich antioxidant contents that maintain the endothelial oxidative status [30]. VCAM-1 is induced on endothelial inflammatory sites [40] and this finding suggests that 5HPO and 10HPO might induce vascular inflammation. The repeated deep frying process is deleterious on the oxidative stability and biochemical characteristics of the oil, in which it generates ROS and other lipid oxidation products. Our earlier experiment [28] reported that heated palm oil increased lipid peroxidation as indicated by a significant increase in serum thiobarbituric acid reactive substance (TBARS). Moreover, the effect of deep frying process on oil thermoxidation has been documented by previous studies [41–44]. ROS in the diet are absorbed into the blood circulation and their increased presence can overwhelm the intercellular antioxidant defence, leading to oxidative stress which can induce endothelial injury [2, 45]. Vascular endothelial cells, which provide a physical barrier for the underlying smooth muscle cells and play a pivotal role in maintaining cardiovascular homeostasis, are sensitive to disturbances in the redox steady state [46]. Previous reports have demonstrated the association between the disturbance in the redox steady state with modulated endothelial function and inflammation [47–50]. Therefore, prolonged intake of 5HPO and 10HPO may induce inflammation, hence endothelial cells respond by expressing VCAM-1.

VCAM-1 is a member of the immunoglobulin gen superfamily that participates in the adhesion and migration of leukocytes into tissues during immune response. We measured the aortic expression of VCAM-1 in rats because VCAM-1 is expressed on endothelial cells in inflammatory sites. Although VCAM-1 has been well documented to be associated with the development of atherosclerosis [51–53], several studies also demonstrated that the expression of VCAM-1 was increased in patients with hypertension [17–19, 54]. A study performed by Parissis et al. [17] showed that hypertensive patients exhibited higher plasma levels of VCAM-1. We found that there was a significant positive correlation between blood pressure and VCAM-1 density on endothelial cells. This suggests that augmented VCAM-1 expression may reflect inflammation and endothelial dysfunction and hence impairing the regulation of blood pressure. This is because a significant reduction in the cell adhesion molecules expression may indicate a better preservation of endothelial function [55]. Besides, we do believe that the increased VCAM-1 (that is inflammation) may play a role, at least in part, in causing adverse remodelling. A previous study documented that increased inflammatory mediator aggravated vascular remodelling [56]. Remodelling impairs the compliance of the blood vessel and leads to pathogenesis of raising blood pressure [57].

In addition, our results suggest that intake of 5HPO and 10HPO is detrimental to vascular morphometry as well as the regulation of blood pressure. Intake of 5HPO and 10HPO increased blood pressure, morphometric alterations, and expression of VCAM-1 significantly than 1HPO. In fact, we found that rats fed 1HPO did not exhibit significant change when we compared to the control and FPO groups. Palm oil contains saturated fatty acids and unsaturated fatty acids at almost equal ratio [58], in which saturated fatty acids are more resistant to thermoxidation. Though being heated once, the palm oil might still retain abundant antioxidants and generate less ROS than 5HPO and 10HPO and hence protects the vascular endothelium from inflammation. Therefore, it suggests that intake of palm oil heated once might not cause remarkable harmful effect in rats. However, when the oil is reused repeatedly, deleterious effects ensue.

This study provides the hints about the pivotal role of inflammation in the blood pressure raising effect of repeatedly heated palm oil. Repeatedly heated palm oil induces inflammation and vascular remodelling in the rats, which subsequently leads to an increase in blood pressure. Further research need to focus on more inflammatory biomarkers such as intercellular adhesion molecule-1 (ICAM-1), endothelin-1, and others as well as their pathways to understand the possible molecular mechanisms involved in the inducing of hypertension by heated palm oil.

## 5. Summary

In conclusion, prolonged intake of repeatedly heated palm oil appears to increase blood pressure in rats, which might be mediated by inflammation and endothelial dysfunction, as reflected by the adverse vascular remodelling and the induction of VCAM-1 expression on endothelial cells.

## Figures and Tables

**Table 1:** Body weight and food intake for all the experimental groups.

	Groups				
	Control	FPO	1HPO	5HPO	10HPO
Food intake (g/week)	163.67 ± 4.60	151.42 ± 4.94	159.18 ± 5.01	153.61 ± 4.031	152.42 ± 4.11
Initial body weight (g)	252.50 ± 7.09	230.67 ± 4.52	230.17 ± 11.65	244.33 ± 10.48	245.33 ± 7.14
Final body weight (g)	485.83 ± 34.25*	477.50 ± 20.35*	440.67 ± 16.96*	503.67 ± 28.23*	504.00 ± 30.66*
Weight gain (g)	233.33 ± 36.38	246.83 ± 20.74	210.50 ± 27.46	259.33 ± 36.75	258.67 ± 36.56

Data are expressed as mean ± SEM. FPO: fresh palm oil; 1HPO: one-time-heated palm oil; 5HPO: five-time-heated palm oil; 10HPO: ten-time-heated palm oil.

*P < 0.05 versus initial body weight for the same group.

**Table 2:** Aortic morphometric measurements.

	Group				
	Control	FPO	1HPO	5HPO	10HPO
Intima-media thickness (*μ*m)	105.26 ± 2.18	107.87 ± 1.38	112.00 ± 5.51	134.54 ± 1.71*	143.09 ± 3.83*
Lumen diameter (mm)	1.33 ± 0.03	1.27 ± 0.04	1.33 ± 0.04	1.39 ± 0.04	1.35 ± 0.04
Intima-media area (mm^2^)	0.48 ± 0.02	0.47 ± 0.01	0.51 ± 0.04	0.65 ± 0.02*	0.67 ± 0.02*
Lamellar units	10.33 ± 0.67	9.67 ± 0.51	10.07 ± 0.28	9.89 ± 0.71	10.58 ± 0.56
CWT (10^4^ dyne/cm)	1.05 ± 0.06	0.97 ± 0.03	1.06 ± 0.04	1.25 ± 0.03*	1.27 ± 0.05*
Tensile stress (10^4^ dyne/cm^2^)	99.88 ± 5.65	89.93 ± 3.04	95.73 ± 3.80	92.88 ± 3.37	89.08 ± 5.05

Data are expressed as mean ± SEM. FPO: fresh palm oil; 1HPO: one-time-heated palm oil; 5HPO: five-time-heated palm oil; 10HPO: ten-time-heated palm oil; CWT, circumferential wall tension.

*P < 0.05 versus control, FPO, and 1HPO.
